# Beyond the Matrix: The Many Non-ECM Ligands for Integrins

**DOI:** 10.3390/ijms19020449

**Published:** 2018-02-02

**Authors:** Bryce LaFoya, Jordan A. Munroe, Alison Miyamoto, Michael A. Detweiler, Jacob J. Crow, Tana Gazdik, Allan R. Albig

**Affiliations:** 1 Biomolecular Sciences PhD Program, Boise State University, Boise, ID 83725, USA; brycelafoya@u.boisestate.edu (B.L.); jacobcrow@u.boisestate.edu (J.J.C.); 2 Department of Biological Sciences, Boise State University, Boise, ID 83725, USA; jordanmunroe@u.boisestate.edu (J.A.M.); mikedetweiler@u.boisestate.edu (M.A.D.); tanagazdik@u.boisestate.edu (T.G.); 3 Department of Biological Science, California State University, Fullerton, CA 92831, USA; almiyamoto@fullerton.edu

**Keywords:** integrin, extracellular matrix, counterreceptor, disintegrin, immune system, stem cell, pathogen, virus, bacteria, venom, growth factor, hormone

## Abstract

The traditional view of integrins portrays these highly conserved cell surface receptors as mediators of cellular attachment to the extracellular matrix (ECM), and to a lesser degree, as coordinators of leukocyte adhesion to the endothelium. These canonical activities are indispensable; however, there is also a wide variety of integrin functions mediated by non-ECM ligands that transcend the traditional roles of integrins. Some of these unorthodox roles involve cell-cell interactions and are engaged to support immune functions such as leukocyte transmigration, recognition of opsonization factors, and stimulation of neutrophil extracellular traps. Other cell-cell interactions mediated by integrins include hematopoietic stem cell and tumor cell homing to target tissues. Integrins also serve as cell-surface receptors for various growth factors, hormones, and small molecules. Interestingly, integrins have also been exploited by a wide variety of organisms including viruses and bacteria to support infectious activities such as cellular adhesion and/or cellular internalization. Additionally, the disruption of integrin function through the use of soluble integrin ligands is a common strategy adopted by several parasites in order to inhibit blood clotting during hematophagy, or by venomous snakes to kill prey. In this review, we strive to go beyond the matrix and summarize non-ECM ligands that interact with integrins in order to highlight these non-traditional functions of integrins.

## 1. Introduction

**Figure 1:**
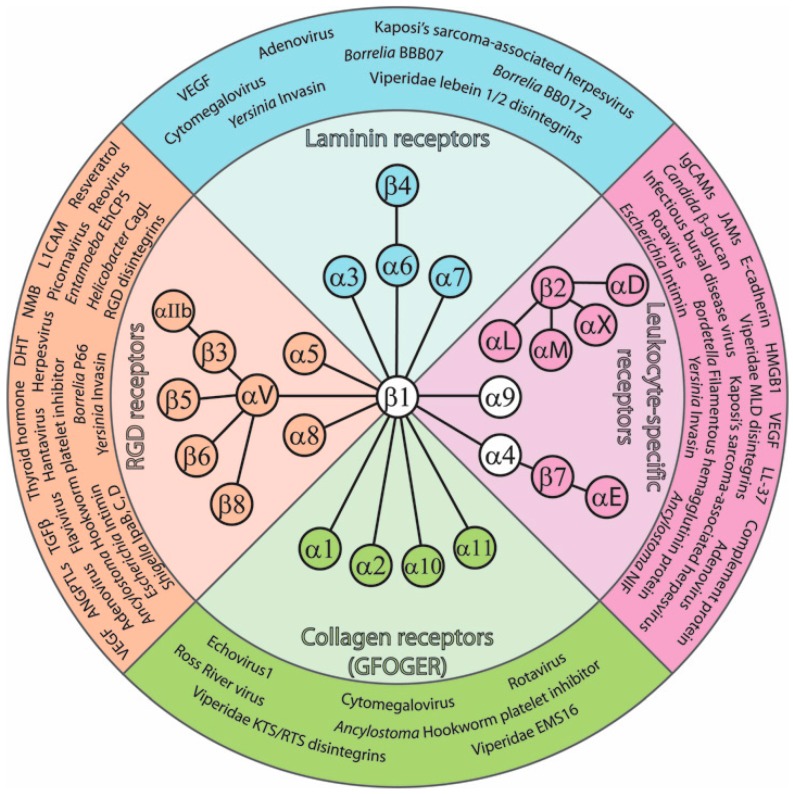
Integrin heterodimers and their ligands. Integrins are heterodimeric cell surface receptors that bind extracellular matrix (ECM) molecules. In addition to this role, integrins also bind many non-ECM ligands. Integrin subunits connected by a ray represent heterodimeric α/β binding partners. The inner ring depicts integrin heterodimers grouped into families based upon their classical binding profile. These families include RGD receptors, collagen (GFOGER) receptors, laminin receptors, or leukocyte-specific receptors. Within the outer ring, the non-ECM ligands of these families are listed. Non-ECM ligands include growth factors, hormones, venomous compounds, disintegrins, bacterial proteins, fungal polysaccharides, viruses, polyphenols, and counterreceptors.

The adhesion of cells to extracellular matrices is a fundamental requirement for multicellular organisms, and animals employ many mechanisms to fulfill this demand. Amongst these mechanisms of adhesion, integrins are perhaps the most ubiquitous. Integrins are heterodimeric transmembrane proteins, made up of non-covalently paired α and β subunits, which serve as adhesion and signaling hubs at the cell surface. In mammals, there are 18 α-integrin subunits and eight β-integrin subunits that can combine to form as many as 24 unique heterodimeric receptor complexes [1]. Typically, ligand binding is carried out through integrin receptor recognition of small peptide sequences. Target sequences for integrins can be as simple as the RGD or LDV tripeptides, or more complex as in the case of the GFOGER peptide [1]. Many classical extracellular matrix (ECM) proteins contain these short integrin recognition motifs. RGD sequences are found in both vitronectin and fibronectin, an LDV motif is present in fibronectin, GFOGER is found within collagen, and the target sequence within laminin has not yet been defined [1]. These sequences are not globally recognized by all integrins; therefore, integrin heterodimers are often grouped by the target sequences they specialize in recognizing (Figure 1). Once bound to its ligand, an integrin not only provides adhesion, but also initiates signaling mechanisms which allow cells to respond to the mechanical and chemical properties of the cellular microenvironment. The primary signaling mediators working downstream of integrins include focal adhesion kinase (FAK), Src-family protein tyrosine kinases, and integrin-linked kinase (ILK) [2]. Upon adhesion, cytoskeletal proteins are recruited to the cytoplasmic tails of integrins, forming a linkage between the ECM and cytoskeleton [2].

As a family of proteins, integrins and many of their downstream signaling intermediates have a long evolutionary history. Beginning at the root of the metazoan lineage, sponges have been shown to express α- and β-integrin subunits [3,4] that bind to peptides in a fashion similar to mammalian integrins [5]. Interestingly, integrin-encoding genes have been found in the single-celled eukaryotic relatives of metazoans, thus the origin of integrins predates the emergence of metazoans [6]. Moreover, components of integrin signaling machinery such as FAK, Src, and ILK, and integrin-interacting cytoskeletal proteins such as α-actinin, talin, vinculin, and paxillin, have pre-metazoan origins [6]. This suggests that integrins and their aforementioned signaling machinery may have played an important role in the evolution of multicellularity.

Beyond their traditional role as mediators of ECM attachment, a vast literature has developed that describes interactions between integrins and ligands that are not located in the classical extracellular matrix. For example, integrins have been shown to interact with various proteins on the surfaces of eukaryotic, prokaryotic, and fungal cells, as well as a range of viruses. Within eukaryotes specifically, integrin-mediated cell-cell adhesion has been shown to coordinate a range of interactions and processes including leukocyte extravasation, stem cell homing, tumor cell migration, erythrocyte development, and interactions in the immune system. For infectious prokaryotes, integrins are exploited as cell surface adhesion receptors that mediate colonization and/or the bypassing of epithelial or endothelial barriers. Beyond mediating cellular interactions, integrins can also serve as cell surface receptors for hormones, growth factors, and polyphenols. Finally, integrins are also common targets for a class of small molecules called disintegrins, which are components of various snake venoms, and are also employed by hematophagous parasites. Collectively, the range of non-ECM molecules that interact with integrins is vast, making integrins indispensable mediators of cell biology at large. The goal of this review is to highlight some of the best understood non-ECM ligands of integrins and discuss the diverse biological roles for these interactions.

## 2. Integrin-Mediated Cell-Cell Interactions

The first integrins discovered were isolated based on their ability to bind to fibronectin, which had itself just recently been identified (reviewed in [7]). However, in the early days of integrin research, several groups studying cell-cell adhesion in the immune system were also on the forefront of integrin identification (reviewed in [8,9]). In fact, integrins that mediate cell-cell adhesion in the immune system were among the first integrins to be characterized [8]. As more integrins were discovered, it became apparent that the majority of integrins established cell-ECM connections rather than cell-cell connections. Nonetheless, it is important to understand that integrins are important mediators of cell-cell adhesion. The term counterreceptor has often been used to describe membrane-bound, non-matrix integrin ligands which facilitate cell-cell contact and will be used to differentiate them from the other non-matrix ligands in this review. While there are many types of counterreceptors, the best-known examples include the immunoglobulin superfamily cell adhesion molecules (IgCAMs) and junctional adhesion molecules (JAMs). Collectively, interactions between integrins and these counterreceptors mediate a range of immune cell functions including leukocyte extravasation from the blood stream, immunological surveillance in the gut, and hematopoietic stem cell homing and mobilization. Additionally, non-ECM ligands enhance the interaction between pathogens and phagocytic immune cells, acting as phagocytic primers and inducers of neutrophil extracellular traps. Beyond the immune system, non-ECM-based integrin interactions are important during the transmigration and metastasis of tumor cells, and during erythrocyte development. Integrins and the non-ECM ligands that mediate these cell-cell interactions are listed in Table 1.

### 2.1. Integrin-Counterreceptor Interactions in Leukocyte Extravasation

**Figure 2:**
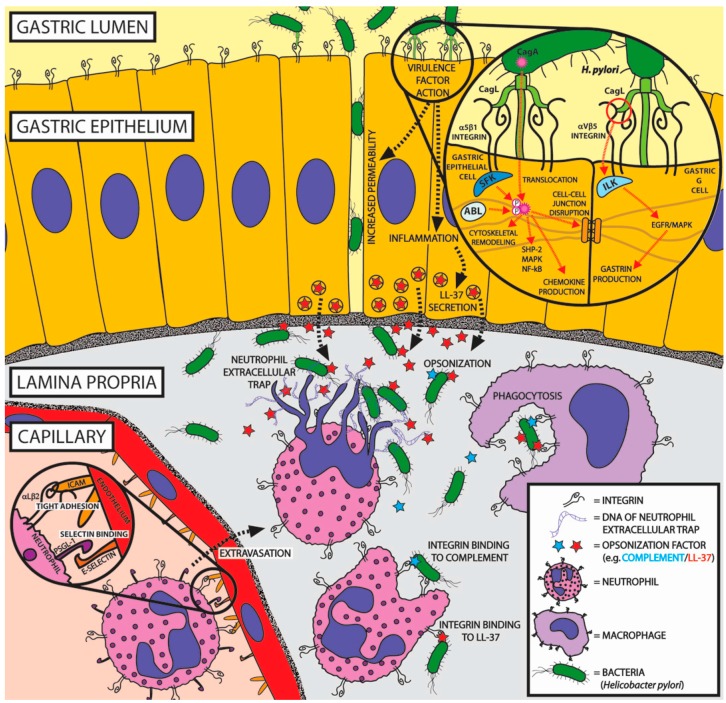
Integrins act as “double agents” during *Helicobacter pylori* infection in the stomach, serving to potentiate bacterial pathogenicity while also aiding in the immune response. *H. pylori* bacteria in the gastric lumen bind integrins on gastric epithelial cells in order to inject the virulence factor CagA. As shown in the magnified view of this process, docking of α5β1 integrin is achieved through integrin affinity for the RGD motif of the CagL protein component of the type IV secretion system (T4SS). Integrin α5β1-mediated stabilization of the T4SS facilitates the translocation of CagA while activating intracellular kinases. Once in the cytosol, CagA is phosphorylated by Src family kinases (SFKs) and Abelson (ABL) kinases, which potentiates its virulence. Phospho-CagA activates Src homology 2 domain-containing phosphatase-2 (SHP-2) and mitogen-activated protein kinase (MAPK) signaling, triggering cytoskeletal remodeling. CagA disrupts cell-cell junctions, activates the nuclear factor-κB (NF-κB) pathway, and stimulates cytokine production. Alternatively, CagL docking with αVβ5 integrin on gastric G cells activates integrin-linked kinase (ILK), which stimulates epidermal growth factor receptor (EGFR) and MAPK activation, inducing gastrin production. These mechanisms increase the permeability of the gastric epithelium, which aids *H. pylori* dissemination into the underlying lamina propria. This stimulates an inflammatory response causing the release of the antimicrobial peptide LL-37 from gastric epithelial cells and recruitment of immune cells from the blood stream. As shown in the magnified view of the recruitment process, leukocytes first stick to inflamed endothelium through selectin binding, which facilitates integrin-mediated tight adhesion. This leads to leukocyte extravasation into the lamina propria, where neutrophils and macrophages phagocytize bacteria. Phagocytosis is mediated through integrin recognition of the opsonization factors LL-37 and complement. Neutrophil extracellular traps (NETs) are stimulated through integrin interaction with pathogens.

Integrin-counterreceptor interactions play multiple roles during extravasation, a process in which white blood cells are recruited from the blood stream to a site of inflammation (depicted in Figure 2). Extravasation begins when glycoproteins on the leukocyte cell surface, such as P-selectin glycoprotein ligand-1 (PSGL-1), bind endothelial selectins, which allows the leukocyte to slow down as it rolls along the vessel wall [33]. Next, local chemokines stimulate leukocyte integrins to adopt a high-affinity state, causing them to bind specific immunoglobulin superfamily cell adhesion molecules (IgCAMs) on endothelial cells [34]. There are many integrin-IgCAM pairs involved in this process: αLβ2 (LFA-1) integrin binds to ICAM1, 2, or 3, αMβ2 (Mac-1) integrin binds to ICAM1, and α4β1 (VLA-4) integrin binds to VCAM1 or MAdCAM1 (reviewed in [10]). Additionally, leukocyte integrins can bind a family of proteins known as junctional adhesion molecules (JAMs) found on endothelial cells. Similar to integrin-IgCAM interactions, integrins display specificity for particular JAM proteins: JAM-A binds to αLβ2, JAM-B binds to α4β1, and JAM-C binds to αMβ2 [11]. All of these integrin-counterreceptor binding events serve to tightly adhere the leukocyte to the endothelium, enabling the white blood cell to cross the endothelial layer (a process known as transendothelial migration) in order to reach the inflamed tissue.

### 2.2. Non-ECM Integrin Ligands as Primers for Phagocytosis

One of the best-characterized examples of non-ECM integrin-binding ligands in the immune system involves the interplay of integrins with the complement system. Complement proteins aid in the immune system’s clearance of pathogens by attaching to invaders and tagging them for destruction. Integrin β2 is essential for complement recognition by the complement receptors αMβ2 (Mac-1, CR3) and αXβ2 (CR4) integrins [23]. αMβ2 and αXβ2 ligation with the iC3b component of complement induces the phagocytosis of complement opsonized pathogens and particles by phagocytic immune cells (depicted in Figure 2) [24]. Despite high homology between both integrins, they bind the iC3b fragment of complement via distinctive receptor sites, which may afford a greater diversity of leukocytes in opsonized target recognition modes [35]. This leads to the intriguing possibility of cooperativity between two integrins binding the same complement molecule [35].

Phagocytosis mediated by integrins is not strictly complement dependent. Human cathelicidin peptide LL-37, an antimicrobial peptide that binds to the prokaryotic cell wall, inserts itself into the membrane, and enhances phagocytosis by interacting with αMβ2 integrin present on neutrophils and macrophages [26,27]. As an important part of innate defenses, LL-37 is expressed in various mammalian tissues and released upon contact with bacterial invaders [29]. For example, upon infection by *Helicobacter pylori*, gastric epithelial cells express and secrete LL-37, thus tagging the bacterial invaders for destruction by phagocytic immune cells (depicted in Figure 2) [28]. Interestingly, LL-37 binds αMβ2 with a comparable strength to complement C3d, a ligand with one of the strongest known affinities for αMβ2 [25].

### 2.3. Non-ECM Integrin Ligands as Triggers for NETosis

Another example of non-ECM integrin ligation at work in the innate immune system is the neutrophil extracellular trap (NET). In the process of NETosis, chromatin is ejected from neutrophils upon interaction with pathogens, thus entangling foreign invaders in a web of DNA and histones (depicted in Figure 2) [36]. This process is mediated through pathogen recognition by neutrophil integrins. For example, the pathogen-associated molecular pattern, β-glucan, found on *Candida albicans* is recognized by αMβ2 at a unique lectin-like domain, and its binding stimulates NETosis [21]. Once stimulated, anti-microbial peptides are integrated into NETs. These include defensins and the αMβ2 ligand LL-37 [22]. NETosis is not exclusively used to trap foreign invaders, as it is also involved in wound healing and sterile inflammation [37]. For instance, during cell necrosis the chromatin protein high-mobility group box 1 (HMGB1 aka amphoterin) is released extracellularly and recruits neutrophils by binding integrin β2 [38]. HMGB1 has been demonstrated to be an inducer of NETosis when presented on platelets during thrombosis [30]. This evidence suggests that HMGB1 serves as a molecule that is capable of signaling to white blood cells the presence of tissue damage through leukocyte integrins. Although αMβ2 plays a starring role in the literature connecting NETosis and integrins, other integrins may be involved. Bacterial invasin proteins from *Yersinia pseudotuberculosis* interact with neutrophil integrin β1, stimulating phagocytosis while also causing the release of NETs [39]. In addition to trapping cells within a tangle of DNA and histones, fibronectin has been identified in NETs, which ligates to αVβ3 and α5β1 integrins found on neutrophils and cancer cells, thus potentially enhancing cancer cell-leukocyte interaction [40]. Collectively, this information demonstrates rich evidence for the importance of integrin engagement during NETosis.

### 2.4. Non-ECM Integrin Ligands in Immune Surveillance

The intestinal immune system must display tolerance towards commensal microbiota and food antigens while still maintaining immunogenicity against pathogens. In the gut mucosa, resident antigen-presenting cells (APCs) have the job of sampling foreign antigens. APCs then transport these antigens to specialized gut-associated lymphoid tissue where they can interact with naïve T cells to promote their maturation. Additionally, the APCs imprint intestinal homing properties on the T cells through inducing the expression of α4β7 integrin and C-C chemokine receptor type 9, a receptor for the gut-associated C-C motif chemokine 25 (CCL25) [41]. Mature T effector cells then reenter the circulation and can be recruited back to the gastrointestinal tract during times of inflammation through gut endothelial expression of CCL25 and the α4β7 counterreceptor, MAdCAM1 [17]. There is also a role for α4β1-VCAM1 interactions in the gut; this pair mediates the binding of effector T cells to inflamed gut epithelium [12].

Integrins in the gut also bind to cadherins to modulate the immune response. For instance, cadherin 26 binding to integrins αE and α4 can lead to a T cell immunosuppression phenotype [42]. Moreover, this study found that a similar phenotype is provoked through the treatment of T cells with a soluble form of cadherin 26. So, unlike the integrin-mediated IgCAM interactions in the gut, cadherin binding appears to moderate the immune response. It has been suggested that this interaction may therefore be involved in resolving inflammation [42]. Cadherin-integrin interactions in the lungs have been shown to mediate the engagement of cytotoxic T lymphocytes (CTLs) with cancer cells. Here, CTLs employ αEβ7 integrin to engage E-cadherin on cancer cell surfaces in order to facilitate accurate targeting and release of cytotoxic granules [20].

### 2.5. Integrin-Mediated Stem Cell Homing

The homing and mobilization of hematopoietic stem cells (HSCs) to and from the bone marrow is also regulated by integrins (reviewed in [13]). After the treatment of hematologic malignancies with large doses of radiation and/or chemotherapy, transplantation of HSCs is commonly performed. Success of the HSC engraftment within the bone marrow is dependent upon proper HSC homing to a bone marrow niche where they can regenerate hematopoietic lineages. New evidence is revealing that integrin engagement of counterreceptors plays a critical role in this homing process. For example, Murakami et al. determined that a subpopulation of murine HSCs expressing integrin β7 have enhanced homing capabilities to bone marrow niches compared to their counterparts which do not express β7 [18]. Mechanistic insight was provided when it was revealed that α4β7 integrins on HSCs were binding MAdCAM1 present on endothelial cells within the bone marrow niche, and β7 knockout HSCs showed decreased CXCR4 homing receptor expression [18].

In addition to α4β7-MAdCAM1 interactions, α4β1-VCAM1 binding also mediates HSC retention in bone marrow niches. The importance of α4 integrin to this interaction is supported by the phenotypes of multiple α4 knockout mouse models that show elevated numbers of HSCs in the bloodstream relative to wild-type littermates (reviewed in [13]). The treatment of mice with Bortezomib, which inhibits the expression of VCAM1, also increases HSC mobilization [14]. Together, these results support a role for integrins in holding HSCs within the bone marrow and have raised great clinical interest in using Bortezomib-induced mobilization for the harvesting of HSCs from the peripheral blood of healthy individuals for use in transplantation.

Another integrin-targeting small molecule antagonist is the drug Firategrast; it inhibits α4β1 and α4β7 activity and can also be used to mobilize HSCs from the bone marrow to the circulation, making HSC harvesting much less invasive. There is particular interest in using Firategrast for in utero hematopoietic cell transplants (IUHCT). These transplants can be especially useful for diseases where a more mature immune system can thwart the therapeutic benefit of the transplanted cells (reviewed in [43]). Firategrast was tested in a mouse model of IUHCT and found to increase long-term engraftment of HSCs; there was 15% engraftment at six months with Firategrast, compared to 3% with vehicle alone [44]. The current thinking is that the mobilization of endogenous HSCs through the disruption of integrin adhesion by Firategrast makes room in the bone marrow for transplanted HSCs to compete with endogenous cells for niche binding. Although still in preclinical studies, Firategrast is well-tolerated by adults but has not yet been tested in children (reviewed in [43]).

Some interesting new data on mesenchymal stem cell (MSC) homing demonstrates that the role of integrin αL (CD11a) in MSC transmigration across vessel endothelium differs from that of leukocyte extravasation [45]. Using zebrafish whose endothelium was labeled with green fluorescent protein as a model system, mammalian leukocytes, cardiac stem cells, and MSCs were transplanted to determine their transmigration properties. As expected, leukocyte extravasation proceeded in an αL-dependent fashion, as αL-blocking antibodies inhibited leukocyte extravasation. However, the blocking antibodies did not inhibit the transmigration of cardiac stem cells or MSCs, indicating that these cells were traversing the endothelium in an αL-independent fashion that was found to rely on the remodeling of the endothelium for vascular expulsion of these types of stem cells. Based on this evidence and additional phenotypic differences in the transmigration of cardiac stem cells and MSCs, the authors have named this alternate process angiopellosis [45].

### 2.6. Integrin-Counterreceptor Interactions in Tumor Cell Migration

Integrin binding to IgCAMs also mediates tumor cell binding to endothelial cells, influencing metastasis. Many of these interactions involve L1CAM (reviewed in [46]); this protein contains an RGD motif that binds to αVβ3 integrin [47,48]. The expression of L1CAM by various types of cancer cells is utilized to engage αVβ3 on endothelial cells. It has been demonstrated that L1CAM expression in glioma tumor cells serves to promote the motility of both cancer cells [31] and endothelial cells [32], thus having important implications for both metastasis and angiogenesis, respectively. Other non-ECM integrin ligands have been implicated in tumor cell migration. For example, when expressed on cancer cells, VCAM1 has been identified as a driver of metastasis due to its ability to bind α4β1 integrin expressed on lymph node endothelium (reviewed in [16]). Additionally, metastatic breast cancer cells express the transmembrane glycoprotein NMB that contains an RGD motif and can bind to α5β1 integrin on adjacent tumor cells. This interaction activates Src and FAK signaling within the tumor and leads to increased growth and metastasis [19].

### 2.7. Integrin-Counterreceptor Interactions in Erythrocyte Development

Since integrins can bind to both ECM and other cells, it is perhaps not surprising that there are modulators that can push integrins towards either a cell-ECM or a cell-cell interaction. During erythrocyte differentiation in the bone marrow, immature erythroblasts cluster around a central macrophage, forming what is known as erythroblastic islands. This cell-cell interaction is mediated by α4β1 on erythroblasts and VCAM1 on macrophages and is an essential part of the maturation process [15]. The same α4β1 integrin can bind to fibronectin in the ECM, and the modulation of α4β1 binding to either macrophages or ECM is in part due to the activity of erythrocyte tetraspanin proteins CD81, CD82, and CD151 [49]. These tetraspanins are co-expressed with α4β1 on human proerythroblasts, where they increase the affinity and/or clustering of integrins to favor α4β1-VCAM1 interactions over α4β1-fibronectin interactions [49].

## 3. Non-ECM Integrin Ligands of Viruses

**Figure 3:**
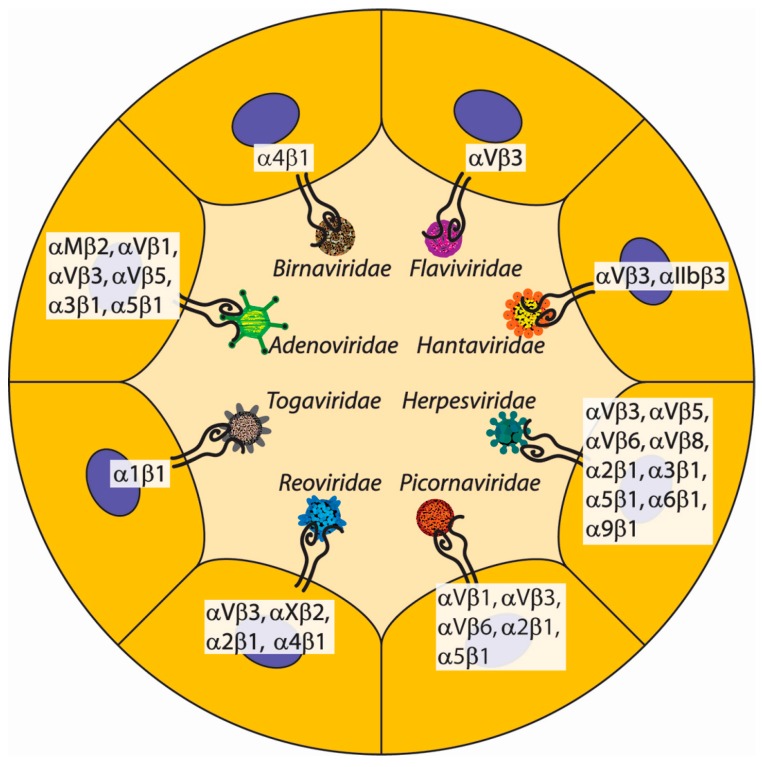
Viruses hijack integrins for adhesion and infectivity. Virus families use specific integrins in order to adhere to target cells for the purposes of internalization and infectivity. Members of the family *Adenoviridae* are non-enveloped viruses with icosahedral capsids that have penton base structures which facilitate RGD-dependent docking with αVβ1, αVβ3, αVβ5, and α5β1 integrins as well as the RGD-independent engagement of α3β1. Adenoviruses also target αMβ2 integrin through an undetermined mechanism. *Birnaviridae* contains members who employ a fibronectin-mimicking IDA peptide to bind α4β1 integrin. Members of the *Flaviviridae* family have an RGD-containing E-protein which binds αVβ3 integrin. Viruses in the family *Hantaviridae* target the plexin-semaphorin-integrin (PSI) domain of αVβ3 and αIIbβ3 integrins. *Herpesviridae* has members that employ a few different mechanisms of integrin engagement for the purposes of viral entry. The envelope protein BMRF-2 contains an RGD sequence that docks α5β1 integrin. The envelope proteins gH and gL dock with αVβ5, αVβ6, and αVβ8. Another envelope protein, known as gB, contains both an RGD motif and disintegrin-like domain, which affords viral targeting of αVβ3, αVβ5, α2β1, α3β1, α6β1, and α9β1 integrins. Members of the *Picornaviridae* family use capsid proteins to target integrins. The targeting of α2β1 integrin proceeds in an RGD-independent manner, while αVβ1, αVβ3, αVβ6, and α5β1 integrins are bound in an RGD-dependent fashion. *Reoviridae* contains members which employ a DGE sequence within a VP4 capsid protein to engage α2β1. Additionally, the reovirus VP7 capsid protein has a GPR tripepetide which recognizes αXβ2, an LDV tripeptide that ligates α4β1, and a novel NEWLCNPDM amino acid sequence that targets αVβ3. *Togaviridae* has members which have a collagen-mimicking spike protein that docks α1β1 integrin.

Although there is debate as to when viruses first emerged in the evolution of life, it is likely that viruses (in one form or another) have co-existed with cells for nearly as long as cells have existed [50]. It is also safe to assume that viruses have a long history of exploiting cell surface receptors to facilitate their infectious cycles. As already discussed, integrins are first present in evolutionary history at the root of the metazoan lineage, and perhaps predate metazoans [3,4,6]. Therefore, it is not surprising that many species of viruses have exploited (and continue to exploit) integrins as a major point of cell attachment, entry, and eventually infection of target cells. A common theme among many of the viruses discussed here is the display of RGD motifs on viral capsids to bind to integrins that are commonly found on either epithelial or endothelial surfaces [51,52]. Presumably, the RGD motif serving as a minimal integrin-binding unit accommodates the viral quest for genomic minimization. Additionally, RGD-recognizing integrins are common in tissues targeted by invading viruses. However, RGD-based mechanisms are not the only means of integrin engagement by viruses, as some viruses employ other integrin targeting motifs. The virus-integrin interactions we have chosen to highlight are in no way an exhaustive list (for a more comprehensive review of the subject, refer to [53,54]). Integrins that participate in viral interactions that we discuss are listed in Table 2 and depicted in Figure 3.

### 3.1. Non-ECM Integrin Ligands of Picornaviridae

Viruses of the *Picornaviridae* family cause a variety of human diseases including aseptic (viral) meningitis, paralysis, hepatitis, and poliomyelitis [87], and there are currently no approved treatments to minimize picornavirus infection. Picornaviruses are non-enveloped viruses with icosahedral capsids, with each face of the 20-sided capsid consisting of three capsid proteins (VP1-3) to form a protomer with 60 subunits. The VP4 protein is contained within the capsid and is thought to help package the single-stranded RNA genome (reviewed in [88]). Several picornaviruses have been shown to exploit integrins as cell surface receptors to facilitate cell invasion. In most cases, the non-ECM ligands that enable picornavirus binding to integrins are located on the VP1-3 capsid proteins.

Members of *Picornaviridae* include the enteric cytopathic human orphan (echo) viruses. Echovirus 1 (EV1) utilizes the α2I functional domain of α2β1 integrin as a docking receptor on the surface of a target cell [56,57]. Although the precise peptide sequence of EV1 that binds to α2β1 integrin has not been discovered, it is known that EV1 binds to the α2I domain of α2β1 integrin 10 times more tightly than collagen [56,89,90]. The structure of the EV1 capsid provides a pentameric arrangement of binding sites for α2β1, which induces the clustering of α2β1 integrins, and is thought to promote the entry of the virus [56]. During infection, EV1 along with α2β1 integrin are taken into the host cell via caveolar endocytosis and moved to a caveosome, where it is thought that the virus ejects its genome into the cytosol [91,92,93,94]. While EV1 utilizes a non-RGD signal to bind its integrin receptor, echovirus 9 (EV9) docks to integrin αVβ3 via an RGD domain located on the EV9 VP1 capsid protein [72]. RGD motifs are also thought to be critical in host cell attachment for echovirus 22 (EV22) to αVβ1 integrins [69,70]. Another member of the *Picornaviridae* family, coxsackievirus A9, utilizes the coxsackievirus and adenovirus receptor (CAR) together with an RGD motif situated in the C-terminal of its VP1 to bind αVβ3 and αVβ6 integrins and gain cellular entry [73,95]. Yet another member of the *Picornaviridae* family, foot-and-mouth disease virus (FMDV), is a major scourge of animal husbandry. The VP1 protein of FMDV has an exposed flexible loop, termed the GH loop, which contains an RGD motif and mediates binding to host α5β1, αVβ3, and αVβ6 integrins [64,74,84,85].

### 3.2. Non-ECM Integrin Ligands of Flaviviridae

*Flaviviridae* is a family of single-stranded, positive sense RNA viruses that are commonly transmitted to human hosts from arthropods such as ticks and mosquitos [96]. Japanese encephalitis virus (JEV), a mosquito-borne member of the genus *Flavivirus*, is a leading cause of viral encephalitis in humans and animals [97,98]. JEV has an envelope protein, called E protein, which contains an RGD motif [99]. Data suggest that JEV utilizes this RGD motif to bind αVβ3 integrin to aid in cellular infection. Specifically, JEV infectivity is reduced by shRNA knockdown of integrin αV and β3 subunits, pretreatment of cells with soluble RGD peptides, or αV/β3 blocking antibodies. Conversely, the expression of β3 integrin promotes infectivity in otherwise resistant cell lines [75]. Finally, the utilization of integrin receptors appears to be a common infection strategy for the *Flaviviridae* family, since other members such as West Nile virus [100,101,102], Murray Valley encephalitis virus [103], dengue virus [104], and yellow fever virus [105] have all been connected with integrin-mediated infection or have at least been demonstrated to possess RGD-containing E proteins.

### 3.3. Non-ECM Integrin Ligands of Herpesviridae

Members of the *Herpesviridae* family of viruses also use integrins for cellular attachment and entry. Epstein-Barr virus (EBV) utilizes α5β1 integrin for infectivity in tongue and nasopharyngeal epithelium by binding host cell integrins with its RGD-containing envelope glycoprotein, BMRF-2 [65]. In addition, the engagement of EBV envelope glycoproteins gH and gL with αVβ5, αVβ6, and αVβ8 integrins induces a conformation in these glycoproteins which facilitates fusion with the target cell membrane [83]. More mechanistic insight is provided by herpes simplex virus (HSV), which also uses gH and gL envelope glycoproteins to dock αVβ6 and αVβ8 integrins, and this engagement routes HSV to acidic endosomes, thus promoting viral entry [86]. Another herpes virus, Kaposi’s sarcoma-associated herpesvirus (KSHV), uses αVβ3 [76], αVβ5 [82], and α3β1 [61] integrins as entry receptors. The expression of the envelope protein, known as glycoprotein B (gB), which is highly conserved across *Herpesviridae* and contains an RGD sequence near its N-terminus, affords KSHV its integrin-binding capacity. However, RGD-mediated binding is not the only mechanism of KSHV-integrin interaction. KSHV glycoprotein B also contains a disintegrin-like domain (DLD) which is capable of binding integrin β1 in an RGD-independent fashion [58]. Walker et al. discovered that α9β1 is the integrin target of the glycoprotein B DLD and plays a critical role in KSHV infection [67]. This mechanism is not unusual among *Herpesviridae* family members, as human cytomegalovirus (HMCV) also uses gB to bind αVβ3, α2β1, and α6β1 through its disintegrin-like domain [58,106].

### 3.4. Non-ECM Integrin Ligands of Togaviridae

Ross River fever is a mosquito-borne disease caused by the Ross River virus (RRV), a member of the *Togaviridae* family. This disease induces arthritis by the viral infection of macrophages within synovial joints [107]. It is believed that the RRV spike protein, known as E2, contains two conserved domains which fold in a manner that mimics collagen IV [55]. This allows for the infection of mammalian cells by docking the collagen receptor, α1β1 integrin, in matrix-binding adherent cell types [55]. 

### 3.5. Non-ECM Integrin Ligands of Adenoviridae

Human adenoviruses, known for causing respiratory, gastrointestinal, and ocular infections, are non-enveloped viruses with icosahedral capsids. At each capsid vertex, a penton base supports a fiber protein [108]. Many adenoviruses require two receptors for efficient infection of cells. The coxsackievirus and adenovirus receptor (CAR) is required for the initial adhesion of adenoviral particles to target cells, while subsequent integrin engagement is required for the internalization of the viral particle [109]. It is the penton base structure that affords adenoviruses a diverse array of integrin targets. RGD peptide sequences are located atop each monomer of the penton base, forming an RGD ring around the fiber protein [78]. The RGD peptides mediate docking to αVβ1, αVβ3, αVβ5, and α5β1 integrins for the purpose of internalization [66,71,110,111,112]. Mechanistically, it is thought that the pentameric structure of the base stimulates integrin clustering and downstream integrin signaling, which further facilitates viral internalization [113,114,115]. Adenoviruses also interact with the laminin binding integrin, α3β1, via its penton base, but in a manner that is RGD-independent [62]. Additionally, αMβ2 integrin on myeloid cells can be targeted by adenoviruses, but this interaction is dictated through an as yet undetermined sequence within the penton base [68].

### 3.6. Non-ECM Integrin Ligands of Hantaviridae

As a member of the *Hantaviridae* family, the rodent-targeting Andes virus can spread to humans through the inhalation of aerosolized excreted virus, targeting human endothelial cells and resulting in several fatal diseases such as hantavirus hemorrhagic fever with renal syndrome and hantavirus pulmonary syndrome [116]. The infection of αVβ3 integrin-expressing endothelial cells occurs through the viral targeting of the PSI domain within the β3 subunit [77]. Interestingly, a human polymorphism that has a leucine to proline substitution at position 33 of the integrin β3 PSI domain was experimentally shown to abolish Andes virus infectivity [77]. Sin Nombre virus also utilizes β3-containing integrins, such as αIIbβ3 and αVβ3, for viral attachment [81]. Using atomic force microscopy (AFM) to study membrane dynamics upon Sin Nombre virus interaction, more mechanistic insight was provided for integrin-dependent hantavirus infectivity. Bondu et al. used AFM data to propose a model in which viral docking to the β3 PSI domain of αIIbβ3, when the integrin is in a low affinity state, enhances integrin *cis* interaction with an RGD-containing G-protein coupled receptor known as P2Y_2_R [117]. This *cis* interaction is thought to induce a switchblade-like conformational change within the integrin that ultimately leads to the endocytosis of the viral bound integrin [117]. Other pathogenic hantaviruses also bind and cause the dysregulation of β3 integrins, resulting in the blockade of endothelial cell migration [118], and the enhancement of vascular endothelial growth factor (VEGF)-mediated vascular permeability [119].

### 3.7. Non-ECM Integrin Ligands of Birnaviridae

Infectious bursal disease virus (IBDV) is an immunosuppressive avian pathogen in the *Birnaviridae* family that attacks the bursa of Fabricius (the site of hematopoiesis in birds) of young chickens, having a major negative impact on the poultry industry. The IBDV capsid is built by 260 trimers of the VP2 polypeptide arranged in an icosahedral lattice [120]. VP2 is the only component of the virus capsid, and contains a conserved, fibronectin-mimicking IDA peptide sequence that binds to α4β1 integrins present on target cell membranes [63]. IBDV binding to α4β1 integrin triggers c-Src tyrosine phosphorylation and actin rearrangement, which creates membrane protrusions that internalize the virus [121].

### 3.8. Non-ECM Integrin Ligands of Reoviridae

The family *Reoviridae* includes the gastrointestinal pathogens, known as the rotaviruses, which are the leading etiological factor of diarrheal disease in young children worldwide [122]. The outer layer of the rotavirus capsid consists of 60 VP4 spike proteins protruding from a VP7 protein shell [123]. It is these outermost structures which mediate host cell binding and infectivity. The VP4 spike protein subunit, VP5, contains a DGE tripeptide sequence that serves to recognize α2β1 integrin on target cells [59,60]. Rotavirus VP7 contains an αXβ2-recognizing GPR tripeptide, as well as an α4β1 ligating LDV motif, embedded in a disintegrin-like domain of the protein [60]. Additionally, rotaviruses can target αVβ3 integrin for the purpose of cellular entry; however, this binding does not occur within the RGD pocket [80]. Rather, it is a novel αVβ3-targeting NEWLCNPDM amino acid sequence within the VP7 protein that is thought to mediate rotavirus-αVβ3 interaction [79]. It has been proposed that reoviruses employ a sequential binding mechanism to multiple receptors for the purpose of internalization. Initial binding to the counterreceptor JAM-A is thought to position the virus for subsequent binding to β1 containing integrins that facilitate internalization [124].

## 4. Non-ECM Integrin Ligands in Venoms

Selectively blocking integrins is a major therapeutic goal when combatting a number of pathologies, and a wide variety of approaches have been initiated. One rich source for anti-integrin compounds are venoms from various snake species [125,126], and the study of venom-derived integrin antagonists remains an active area of research. A venom is defined as a secreted toxin, produced by various types of animals, which is injected into another animal for the purpose of defense or predation. The Viperidae family of snakes (collectively known as the vipers) produce a venom which causes local necrosis and blood coagulation within their prey. The discovery of small integrin-targeting peptides found in the venom of these snakes initiated the study of disintegrins. These small molecular weight (40–100 amino acids in length), non-enzymatic proteins were originally characterized by their platelet-disrupting properties through antagonistic targeting of αIIbβ3 integrin [127]. Since the identification of the first disintegrins, the field has grown with the discovery of many more examples. As discussed below, major families of venom-derived disintegrins include the RGD, MLD, PIII, and KTS/RTS disintegrins. On the other hand, C-type lectin-like proteins are an example of non-disintegrin toxins, which also disrupt integrin activity. Integrin-targeting venomous compounds are summarized in Table 3.

The RGD family of disintegrins is the largest family, although RGD amino acid sequences are not strictly required to be members in this family. Instead, disintegrins containing RGD or similar amino acid motifs, such as KGD, MGD, VGD, and WGD, are all capable of targeting RGD-binding integrins, serving to disrupt their physiological functions. Moreover, not all RGD disintegrins target RGD-binding integrins exclusively. For example, lebein1 and lebein2 are two RGD-containing disintegrins found in the venom of *Macrovipera lebetina*, which have the unusual property of targeting the laminin-binding integrins α3β1, α6β1, and α7β1 in an RGD-independent fashion [133]. They are thought to mimic the integrin-binding motif of laminin, thus allowing these molecules to disrupt cellular attachment to the laminin-rich basement membrane [133].

Other disintegrin families include the MLD-, PIII-, and KTS/RTS-containing disintegrins. Whereas the RGD family of disintegrins possesses an RGD tripeptide (or similar motif) within the integrin-binding loop of the protein, the MLD motif is found at this same position in MLD-containing disintegrins [128]. These MLD disintegrins appear in heterodimeric complexes and are highly dependent on adjacent sequences to target the α4β1, α4β7, and α9β1 leukocyte specific receptor family of integrins [128]. PIII class disintegrins are large multi-domain toxins (60–100 kDa) which use an ECD integrin-targeting tripeptide and contain a metalloprotease domain which is a close homolog to the ADAM (a disintegrin and metalloprotease) family of metalloproteases [150]. The disintegrin known as alternagin uses an ECD tripeptide motif to target α2β1 integrin and disrupt matrix binding [151]. Once bound, alternagin uses its protease domain to cleave β1, causing integrin shedding and further disruption of collagen-induced platelet aggregation [152]. Finally, the KTS/RTS group of disintegrins, found in Viperidae venom, are monomeric proteins which bind the collagen receptor α1β1 integrin [129]. This high level of specificity is not matched by RGD and MLD disintegrins, as KTS/RTS disintegrins only target α1β1 integrin [128].

Another class of toxin found in Viperidae venom is the C-type lectin-like proteins (CLPs). These proteins do not exhibit the sugar-binding capabilities of C-lectin proteins, but instead target collagen-binding integrins [153]. The viper species *Echis carinatus multisquamatus* produces EMS16, a potent and selective inhibitor of α2β1 integrin [130]. X-ray crystallography reveals that EMS16 spatially blocks collagen-integrin ligation through docking with the α2I domain of α2β1 integrin and stabilizing a low matrix affinity integrin conformation [131]. Several studies have shown that many viper-derived CLPs target endothelium and block angiogenesis [130,154,155], while applying CLPs to cancer cells can inhibit cell-collagen binding [153] and metastasis [156]. Integrins that interact with CLPs are summarized in Table 3.

## 5. Bacterial Use of Non-ECM Integrin Ligands

For many bacterial cells, successful adhesion to host cell surfaces is a prerequisite for successful colonization and/or infection. Many bacteria take advantage of the binding capabilities of integrins on cell membranes for infectious purposes. Some bacteria utilize specific integrin dimers for cellular binding, while others exploit extracellular fibrous proteins that naturally bind to integrins for the purpose of translocating virulence factors. For this review, we will highlight three of the most commonly studied interactions between bacteria and integrins. There are other notable examples of bacterial cells using integrins as host cell receptors that we will not discuss: the intimin protein of *Escherichia coli* that binds to α4β1 and α5β1 integrins [157], the IpaB, C, and D proteins of *Shigella flexneri* that bind to α5β1 integrin [158], and the filamentous hemagglutinin protein of *Bordetella pertussis* that binds to αMβ2 integrin [159]. Integrins that participate in bacterial interactions are listed in Table 4.

### 5.1. Non-ECM Integrin Ligands of Borrelia burgdorferi

The spirochete *Borrelia burgdorferi* is the causative agent of Lyme disease, a devastating disease of the nervous system. The natural reservoir for *B. burgdorferi* includes mice, birds, and lizards [160]. These spirochetes are transmitted to humans via tick vectors of the *Ixodes* genus [160]. Once injected into the blood stream, *B. burgdorferi* spirochetes adhere to the microvasculature, transmigrate through the endothelium, and disseminate into various tissues [161]. Characterizing the proteins that enable this pathological mechanism illustrates several interesting examples of how microbes take advantage of host integrins.

A variety of screening techniques have identified at least 19 *B. burgdorferi* proteins that mediate or enhance adhesion to target cells [162]. The majority of these proteins mediate indirect adhesion to mammalian cells via interactions with various ECM molecules. Three proteins however, P66, BBB07, and BB0172, have been shown to interact with integrins on platelets and a variety of cells such as endothelial cells. Prior to the discovery of the P66 protein, it had been known for some time that *B. burgdorferi* cells could adhere to β3 chain-containing integrins [163,164]. The P66 protein was later identified by phage display and shown to bind αVβ3 and αIIbβ3 integrins [165]. P66 displays no typical integrin-binding sites [165], although the adhesion of P66 to integrins can be blocked by soluble RGD peptides, suggesting that P66 may bind into the RGD pocket of β3 integrins [166]. Moreover, a minimal seven-amino acid sequence (QENDKDT) from P66 was found to bind integrins, and the deletion of the aspartic acid residues from this peptide eliminated P66 integrin binding [167]. Despite the integrin-binding activity of P66, the deletion of P66 does not appear to affect *B. burgdorferi* adhesion to microvasculature, a key step proceeding tissue invasion [168]. Instead, the P66 protein (presumably via its integrin-binding activity) appears to be essential for the endothelial transmigration and dissemination of *B. burgdorferi* spirochetes into host tissues [167,168]. Although P66 deletion did not affect microvascular adhesion, *B. burgdorferi* binding to various cells can be blocked by soluble RGD peptides [163], suggesting the presence of other integrin-binding proteins. In support of this, two additional integrin-binding outer membrane surface proteins, BBB07 and BB0172, have been detected on *B. burgdorferi* [169,170]. Although both BBB07 and BB0172 have been shown to interact with α3β1 integrins, only BBB07 contains an RGD motif [170]. Currently, there is little known about the function of α3β1 integrins in endothelial biology, although it has been proposed that α3β1 binding to Laminin 511 in the basal lamina may be linked to endothelial barrier function [171], which could provide a link to the transendothelial migration of *B. burgdorferi* during infection.

### 5.2. Non-ECM Integrin Ligands of Helicobacter pylori

*Helicobacter pylori* infects roughly half of the world’s human population and shares responsibility for gastric complications including stomach ulcers and gastric adenocarcinoma through its infection of gastric epithelial cells [172,173,174]. *H. pylori* utilizes a type IV protein secretion system (T4SS) involving the cytotoxin-associated gene L (CagL) adhesion tip protein to infect target cells with the virulence factor, cytotoxin-associated gene A (CagA) [175,176]. The efficiency of CagA injection is enhanced by an RGD domain present on the CagL protein [177]. CagL interacts primarily with α5β1 integrin; however, αVβ3, αVβ5, and αVβ6 have also been implicated [178,179,180,181]. Interestingly, while the CagL RGD domain is necessary for CagA injection, additional CagL sequences have been identified that enhance integrin binding. For example, an RGD helper sequence, FEANE, is located in close proximity to the RGD domain of CagL and reinforces integrin engagement [177]. Additional domains on CagL that enhance RGD binding include a TSPSA sequence [182], an LXXL sequence that is directly adjacent to the RGD domain [181], and a TASLI sequence located opposite the RGD domain in the CagL integrin-binding domain [182]. CagL-α5β1 interaction leads to the activation of the kinases Src and FAK [179], followed by subsequent tyrosine phosphorylation of the CagA EPIYA amino acid motifs by Src and ABL kinases [183]. These phosphorylation events potentiate CagA pathogenicity (reviewed in [184]). Phospho-CagA interacts with Shp-2 while initiating mitogen-activated protein kinase (MAPK) signaling, and inducing cytoskeletal rearrangements which serve to cause an elongation of epithelial cells and enhance their mobility. CagA also disrupts cell-cell junctions while triggering an inflammatory response, including nuclear factor-κB (NF-κB) activation and chemokine production. Additionally, in a negative feedback loop phospho-CagA downregulates Src activity, ensuring that a reservoir of nonphospho-CagA remain in the cell, which is necessary for a prolonged infection. As mentioned previously, CagL is capable of interacting with other integrins. Interestingly, a novel mechanism of CagL-αVβ5-induced production of gastrin has been uncovered. It was found that CagL ligation to αVβ5 on gastric epithelial cells activates ILK, which in turn activates the epidermal growth factor receptor (EGFR) and subsequently MAPK pathways, serving to induce gastrin expression [178]. This mechanism may explain *H. pylori* induced hypergastrinemia, which is a major risk factor for gastric adenocarcinoma. The integrin-dependent mechanisms of *H. pylori* infection discussed here are depicted in Figure 2.

### 5.3. Non-ECM Integrin Ligands of Yersinia

The Gram-negative bacteria *Yersinia enterocolitica* and *Yersinia pseudotuberculosis* commonly cause foodborne illnesses. These *Yersinia* species express two adhesion proteins that facilitate cellular attachment and invasion of target cells in the small intestine. The *Yersinia* adhesion A (YadA) protein indirectly binds to integrins via interaction with various molecules of the ECM, but is dispensable for cellular invasion [185,186]. However, the *Yersinia* invasin protein directly binds to a variety of β1 subunit-containing integrins (α3, α4, α5, α6, αV) and is crucial for cellular adhesion and invasion [187,188]. *Yersinia* species invading through the small intestine target the apical membrane of Peyer’s patch M-cells, which express integrin β1 [189,190]. Invasins lack the typical RGD domain used to bind integrins, although RGD peptides prevent invasin binding to β1 integrins [191]. This suggests that invasin proteins interact with the RGD binding domain of β1-containing integrin heterodimers. In support of this, the structural analysis of the invasin protein, and comparison to fibronectin, reveals similar structures with key conserved integrin-binding residues, suggesting the convergent evolution of invasins to match fibronectin [192].

## 6. Protists and Multicellular Parasites That Use Non-ECM Integrin Ligands

A broad array of examples of non-ECM ligands for integrins are employed by many parasitic organisms. Here we discuss just a few examples, including non-ECM ligands produced by the amoebozoa *Entamoeba histolytica* and a range of hematophagic (blood-sucking) organisms. These examples illustrate the importance of non-ECM ligands to parasitic infections. Although compared to bacteria and viruses, there is far less literature on the subject of non-ECM ligands as components of pathogenicity in protozoan and multicellular parasites. Non-ECM integrin ligands derived from parasitic organisms are summarized in Table 4.

### 6.1. Non-ECM Integrin Ligands of Entamoeba histolytica

*Entamoeba histolytica* (Eh) causes amoebic dysentery and liver abscess [205] and is responsible for ~100,000 deaths/year [206]. Eh invasion into host tissues involves multiple integrin-mediated steps. The best-characterized of these integrin-mediated steps involves the Eh cysteine protease 5 (EhCP5) binding to αVβ3 integrins [203,204]. The binding of EhCP5 to αVβ3 integrins on colonic epithelial cells via an RGD domain triggers NF-κB-mediated inflammation [203] and mucin exocytosis [204]. The EhCP5 protein has also been shown to interact with α5β1 integrins to mediate local inflammation, which is crucial to Eh invasion into host tissues [201]. Additional involvement of integrins in Eh invasion has been linked to β2 integrin activation and release of reactive oxygen species [207,208] as well as an integrin β1-like receptor present on Eh trophocytes that mediates adhesion to host fibronectin [209].

### 6.2. Non-ECM Integrin Ligands of Hookworms

The hookworm platelet inhibitor (HPI) protein illustrates another fascinating example of non-ECM integrin ligands. Hookworms are blood-feeding intestinal parasites and a leading cause of iron deficiency in humans. HPI was isolated from the hookworm *Ancylostoma caninum* based on its ability to inhibit the function of integrins αIIbβ3 and α2β1 [193,194]. HPI appears to block platelet aggregation and blood clothing, thus enabling continued feeding. Interestingly, sequence and structural analysis has failed to identify any integrin-binding domains in the HPI protein [210]. In addition to the HPI protein, *Ancylostoma caninum* also expresses the neutrophil inhibitor factor (NIF) that interacts with αMβ2 integrins present on neutrophils [202,211]. NIF disrupts αMβ2 interaction with ICAM1 [202], which is necessary for stable neutrophil adhesion to the endothelium and transendothelial migration, thus suppressing local inflammation. Collectively, the combined actions of HPI and NIF help ensure that hookworms are able to feed from their host for a prolonged period of time.

### 6.3. Non-ECM Integrin Ligands of Blood-Sucking Parasites

In addition to *Entamobea histolytica* and *Ancylostoma caninum*, several other examples of integrin inhibition by hematophagic (blood-sucking) animals have been described in the literature (reviewed in [212]). Many of these strategies involve non-ECM integrin ligands that interfere with various integrin-mediated steps that are essential for blood coagulation. The majority of these non-matrix ligands block platelet αIIbβ3 integrin interactions with fibrin, von Willebrand factor, and vitronectin, which are collectively essential for blood coagulation. Many of these integrin disrupting molecules are found in the saliva of hematophagic organisms and not only inhibit platelet aggregation, but also disrupt neutrophil function and angiogenesis [212]. Examples of these integrin disrupting proteins include the decorsin protein from the leech *Macrobdella decora* [195], the vasotab TY and tablysin-15 proteins from the horsefly *Tabanus yao* [196,197], and the disagregin (*Ornithodoros moubata*), YY-39 (*Ixodes pacificus* and *Ixodes scapularis*), and variabilin (*Dermacentor variabilis*) proteins from ticks [198,199,200]. Many of these proteins contain RGD or similar integrin-binding amino acid motifs (KGD, VGD, MLD, KTS, RTS, WGD, or RED) which bind to and interfere with αIIbβ3 integrin function on platelets. Additional RGD or RGD-like integrin antagonists have been identified in silico from other blood-sucking arthropods such as mosquitos and sand flies [212], but have yet to be explored.

## 7. Hormones, Small Molecules, and Growth Factors That Mimic Integrin Ligands

**Figure 4:**
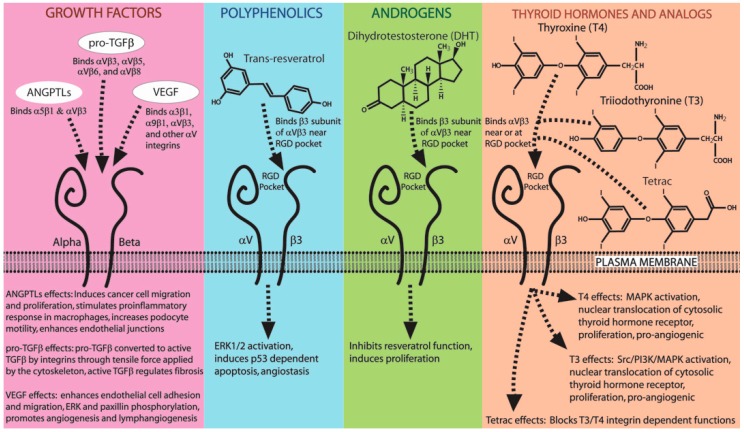
Integrins serve as cell surface receptors for growth factors, hormones, and small molecules. Various growth factors use integrins as cell surface receptors. Angiopoietin-like proteins (ANGPTLs) bind α5β1 and αVβ3 integrins to facilitate a host of cellular effects. Pro-TGFβ is activated by αVβ3, αVβ5, αVβ6, and αVβ8 through the integrin-dependent dissociation of an RGD-containing latency-associated peptide (LAP), thus converting it to its active form. Activated TGFβ acts as a master regulator of fibrosis, among other roles. Vascular endothelial growth factor (VEGF) ligates α3β1, α9β1, αVβ3, and other αV-containing integrins, resulting in cellular effects that promote angiogenesis and lymphangiogenesis. The polyphenol trans-resveratrol, which is derived from grapevines, binds the β3 subunit of αVβ3 integrin near the RGD recognition pocket. This binding event induces extracellular signal-regulated kinase (ERK) activation and p53-dependent apoptosis, while promoting angiostasis. Like trans-resveratrol, the active form of testosterone (DHT) also binds the β3 subunit of αVβ3 integrin near the RGD pocket. DHT-αVβ3 interaction inhibits trans-resveratrol-induced effects and stimulates cellular proliferation. The thyroid hormones, T3 and T4, utilize αVβ3 integrin as a cell surface receptor to activate a range of signaling molecules which induce angiogenesis. When binding to αVβ3 integrin, the thyroid hormone analog tetrac blocks T3/T4 integrin-induced effects.

To this point, we have focused on the non-ECM integrin ligands utilized by various organisms to mediate adhesion to target cell membranes. However, as it turns out, a wide variety of small molecules (including hormones and growth factors) can also interact with integrins, thus broadening the role for integrins in non-ECM interactions. As described in the examples below, integrins binding to small molecules serve a number of cellular functions ranging from cell surface receptor-signaling roles, as in the case of thyroid hormone, dihydrotestosterone, angiopoietin-like proteins (ANGPTLs), and VEGF, to the activation of growth factors, as in the case of TGFβ. Integrins that interact with hormones, small molecules, or growth factors are summarized in Table 3 and depicted in Figure 4.

### 7.1. Small Molecules and Hormones That Bind Integrins (Resveratrol, Thyroid Hormone, DHT)

Trans-resveratrol is a stilbenoid produced in plants such as grapevines that is well-known for its anti-inflammatory activity [213], anti-angiogenic function [214], and anticancer properties [215,216,217]. Resveratrol binds the extracellular portion of the β3 monomer of αVβ3 integrin near the RGD pocket [137]. This binding inhibits αVβ3 integrin-dependent endothelial cell adhesion to vitronectin-coated plates, while also exhibiting angiostatic function and inhibiting tumor growth [139]. Resveratrol binding to αVβ3 integrin induces extracellular signal-regulated kinase (ERK1/2) activation, which leads to p53-induced apoptosis in various cancer cell lines [137,138]. This evidence implicates resveratrol binding αVβ3 integrin as being at least in part responsible for resveratrol’s ability to mitigate angiogenesis and tumorigenesis.

Integrin αVβ3 bears a receptor site for the thyroid hormones T3 and T4 as well as thyroid hormone analogs (reviewed in [140]). Perhaps the first evidence of this interaction was uncovered when Hoffman et al. [218] used an αVβ3 inhibitor (SB-273005) to block T4-induced bone resorption in rats. The binding of T3 and T4 to αVβ3 integrin induces cell proliferation and angiogenesis through MAPK activation, and this effect is negated by a T4 derivative tetraiodothyroacetic acid (tetrac), RGD peptide, and αVβ3 integrin-blocking antibodies, suggesting that the thyroid hormone receptor site is at or near the RGD binding pocket [141,142,143]. Through radioligand binding experiments, it was shown that purified αVβ3 integrin binds T4 preferentially over T3, and binds T4 with high affinity, having a dissociation constant (K_d_) of 333 pM and an EC_50_ of 371 pM [142]. Lin et al. proposed a model for the thyroid hormone receptor activity of αVβ3 integrin that describes two distinct thyroid hormone binding sites on αVβ3 [219]. The site known as “site 1” appears to bind T3 but not T4, while another site called “site 2” binds both T3 and T4 [219]. T3 binding at site 1 leads to Src and phosphatidylinositol 3-kinase (PI3K) activation, which induces nuclear translocation of thyroid hormone receptor (TR) α1, and these effects can be disrupted through the addition of RGD peptide [219]. Meanwhile, T3/T4 binding at site 2 induces ERK activation, which causes nuclear translocation of TRβ1, and only T4-induced effects at this site are disrupted by RGD peptides [219]. This suggests that αVβ3-dependent thyroid hormone signaling acts as a complex, hierarchical system capable of mediating distinct site-specific activities. Since some of these activities are disrupted through RGD binding, and this leads to the possibility that cells embedded in an RGD-rich matrix may respond differentially to thyroid hormone compared to those embedded in an RGD-deficient matrix. Perhaps this is a mechanism by which a ubiquitous receptor, such as αVβ3, can provide tissue-specific responses to thyroid hormone.

In addition to thyroid hormones, αVβ3 integrin also interacts with the biologically active form of testosterone, dihydrotestosterone (DHT). Whether or not this interaction is involved in the normal physiological roles of DHT is unknown; however, DHT binding to αVβ3 has been implicated in cancer cell growth. For example, DHT binding to αVβ3 stimulates MDA-MB-231 breast cancer cell proliferation [143]. Additionally, DHT binding to αVβ3 integrin inhibits resveratrol-induced, p53-dependent apoptosis effects in MDA-MB-231 cells [144], thus highlighting the complexity of hormone signaling through αVβ3 integrin. Through these examples, it is clear that αVβ3 integrin has diverse receptor activity which affords hormones additional non-canonical signaling capacity.

### 7.2. Growth Factors That Bind Integrins (ANGPTLs, TGFβ, VEGF)

Many growth factors are capable of binding integrins. An interesting example is the angiopoietin-like proteins (ANGPTLs), also known as angiopoietin-related proteins (ARPs), which consist of a family of proteins that display structural similarity to the growth factor angiopoietin, although they do not bind classical angiopoietin receptors [220]. Instead, ANGPTLs have been demonstrated to bind various integrins through a C-terminal fibronectin-like domain containing a conserved RGD sequence [221]. In human prostate cancer (LNCaP) cells, ANGPTL2 binds α5β1 integrin, inducing migration and proliferation, and this effect can be negated by use of integrin-blocking antibodies [134]. Furthermore, ANGPTL2 binding α5β1 integrin on macrophages mediates pro-inflammatory responses in mice, and ANGPTL2 knockout mice have muted immune responses, leaving them more susceptible to infections [135]. In the kidney, glomerular podocyte motility is enhanced through cytoskeletal rearrangement induced by ANGPTL3 binding podocyte αVβ3 integrin [145]. The deletion of ANGPTL3 can reduce proteinuria in mouse models of nephropathy, and ANGPTL3 activation of integrin β3 has been identified in patients with nephrotic syndrome [222]. The ANGPTL family also affects vascular integrity. In response to decreased albumin levels during peak proteinuria, podocytes and extrarenal tissues secrete ANGPTL4 into the blood, which binds glomerular endothelial αVβ5 integrin and serves to reduce proteinuria [147]. This effect may be explained by another study where surface plasmon resonance and proximity ligation assays were used to discover that ANGPTL4 also binds another endothelial integrin, αVβ3, which serves to recruit Src kinase and enhance endothelial junction stability, thereby reducing vascular permeability [146]. Taken collectively, these studies suggest that ANGPTL3 binding podocyte integrins enhances proteinuria, whereas ANGPTL4 binding glomerular endothelial integrins decreases proteinuria. The ANGPTLs are a good example of a protein family that mimics a classical extracellular matrix protein in order to bind integrins and implement their cellular effects.

Integrins also play a critical role in the activation of TGFβ (reviewed in [148]). An inactive form of TGFβ (pro-TGFβ) is secreted from cells with an RGD containing latency-associated peptide (LAP) non-covalently bound to TGFβ, which must be removed before TGFβ is biologically active. While the RGD binding αVβ6 integrin plays a key role in separating LAP from TGFβ, other αV-containing integrins, including αVβ3, αVβ5, and αVβ8, have been implicated in this process. Mutation of the LAP integrin-binding site in mice yields normal levels of pro-TGFβ, but results in a lethal phenotype which appears identical to TGFβ deletion [223]. LAP separation is mediated by a tensile force generated by a cell’s cytoskeleton that is transmitted via αVβ6 integrin in order to reshape and activate the pro-TGFβ [149]. The dependence of pro-TGFβ on αVβ6 for activation, and the fact that TGFβ is a well-known master regulator of fibrosis [224], has led to the suggestion that the inhibition of αVβ6 integrin binding may represent a clinical strategy to treat diseases characterized by fibrosis, such as scleroderma [225]. This idea is supported by observations showing that αVβ6 knockout mice [226] or treatment with αVβ6 blocking antibodies [227,228] substantially decrease fibrosis in mouse models of lung fibrosis.

The vascular endothelial growth factors (VEGFs) comprise a group of cytokines which are important mediators of angiogenesis and lymphangiogenesis. VEGF signaling functions through VEGF binding to a group of receptor tyrosine kinases, known as VEGF receptors (VEGFRs). Since this pathway is an inducer of angiogenesis, it has been the target of many anticancer therapies with the hope of inhibiting tumor vascularization. One therapeutic strategy involves inhibiting VEGF-VEGR binding through the targeting of VEGFRs with monoclonal antibodies [229]. However, this approach has not proven as effective as drug developers and clinicians envisioned [229,230]. One reason for this failure may be that VEGFRs are not the only membrane-bound receptor of VEGFs, as these growth factors are also known to bind integrins. Some VEGF isoforms are integrated into the extracellular matrix, where they bind α3β1, αVβ3, and other αV integrins to promote endothelial cell adhesion [132]. Interestingly, the solubility of VEGF ligands greatly effects the integrin response. Vlahakis et al. found that when α9β1 integrin binds immobilized VEGF-A, it induces the recruitment of VEGFR2 into macromolecular structures at the cell membrane [136]. This serves to permit endothelial cell adherence and migration on VEGF-A functionalized Petri dishes, and stimulates the phosphorylation of the downstream effectors paxillin and ERK [136]. In contrast, when soluble VEGF binds α9β1 integrin, paxillin is phosphorylated, but neither the phosphorylation of ERK nor the formation of VEGFR2 macromolecular complexes are induced [136]. Moreover, VEGF-A is not the only VEGF member to have these functions. VEGF-C and VEGF-D also bind α9β1 integrin, stimulating the phosphorylation of paxillin and ERK, while contributing to lymphangiogenesis [231]. Taken together, these findings suggest a VEGF-induced synergy between VEGFR and integrins. Therefore, it may be beneficial to co-target integrins when employing an anti-VEGF therapeutic strategy during cancer treatment.

## 8. Conclusions

Throughout this review, we have sought to venture beyond the matrix and highlight biological examples of integrin ligands that do not fit the classical model of ECM-mediated integrin function. Given the strong conservation of integrins across much of the biological world, it is no surprise that there exists an extremely diverse array of these non-ECM integrin ligands. Consequently, interactions between integrins and non-ECM ligands are actively being exploited for a number of applications in the biotechnology realm. RGD peptides are being used to target liposomes and small molecules to specific tissues for various purposes, including the improvement of chemotherapeutic delivery to cancer cells [232,233,234]. Similarly, RGD peptides are also being used to target viral particles to various tissues. For instance, the new field of “chemical virology” seeks to load viral capsids with chemotherapeutics that, in some instances, utilize RGD functionalization to deliver these nanoparticles to specific tissues [235]. In a related example, a plant virus known as the cowpea mosaic virus, which does not normally target mammalian cells, was functionalized with RGD peptides to successfully target cancer cell lines [236]. Demonstrating another example of applied integrin biotechnology, various artificial “extracellular matrices” are now being created and designed with incorporated RGD peptides to enable cell seeding and growth [237]. Two exciting examples include the development of graphene that has been functionalized with RGD peptides, which is being used to detect nitric oxide release from living cells [238], and DNA origami tubes that have been tagged with RGD peptides and shown to bind neural stem cells and promote their differentiation [239]. These instances and many others provide fascinating examples of how the unique binding properties of integrins continue to be uncovered and utilized.

## Figures and Tables

**Table 1:** Selected non-ECM ligands which mediate cell-cell interactions.

Integrin Dimers	Common Name		Non-ECM Ligand	Function of Interaction [Key Refs]
α4β1	VLA-4	Very late antigen-4	MAdCAM1 VCAM1 AM-B	Leukocyte adhesion [10,11,12] Leukocyte adhesion [10,11,12] Erythrocyte differentiation [13,14,15] Cancer cell metastasis [16] Leukocyte transmigration [11]
α4β7	LPAM	Lymphocyte Peyer’s patch adhesion molecule	MAdCAM1	T-lymphocyte homing [17] HSC homing to bone marrow [18]
α5β1	Fibronectin receptor	Fibronectin receptor	Glycoprotein NMB	Cancer cell growth, metastasis [19]
αEβ7			E-cadherin	Cytotoxic T cell targeting of tumor cells [20]
αLβ2	LFA-1	Lymphocyte function associated antigen-1	ICAM1, 2, 3 JAM-A	Leukocyte adhesion [10,11] Leukocyte transmigration [11]
αMβ2	Mac-1/CR3	Macrophage antigen-1/Complement receptor-3	ICAM1 β-glucan Complement C3 LL-37 JAM-C HMGB1	Leukocyte adhesion [10,11] NETosis [21,22] Phagocytosis [23,24] Bacterial opsonization [25,26,27,28,29] Leukocyte transmigration [11] NETosis [30]
αVβ3	Vitronectin receptor	Vitronectin receptor	L1CAM	Cancer cell metastasis [31,32]
αXβ2	CR4/CD11c/CD18	Complement receptor-4	Complement C3	Phagocytosis [23,24]

mucosal addressin cell adhesion molecule (MAdCAM), vascular cell adhesion molecule (VCAM), intercellular adhesion molecule (ICAM), junctional adhesion molecule (JAM), glycoprotein non-metastatic gene B (Glycoprotein NMB), high mobility group box protein (HMGB), L1 cell adhesion molecule (L1CAM), hematopoietic stem cell (HSC), neutrophil extracellular trap (NET).

**Table 2:** Selected integrin binding by viruses.

Integrin	Virus Name [Key Refs]
α1β1	Ross River virus [55]
α2β1	Echovirus 1 [56,57] Cytomegalovirus [58] Rotavirus [59,60]
α3β1	Kaposi’s sarcoma-associated herpesvirus [61] Adenovirus [62]
α4β1	Infectious bursal disease virus [63] Rotatvirus [60]
α5β1	Foot-and-mouth disease virus [64] Epstein-Barr virus [65] Adenovirus [66]
α6β1	Cytomegalovirus [58]
α9β1	Kaposi’s sarcoma-associated herpesvirus [67]
αMβ2	Adenovirus [68]
αVβ1	Echovirus 22 [69,70] Adenovirus [71]
αVβ3	Echovirus 9 [72] Coxsackievirus A9 [73] Foot-and-mouth disease virus [74] Japanese encephalitis virus [75] Kaposi’s sarcoma-associated herpesvirus [76] Cytomegalovirus [58] Andes virus [77] Adenovirus [78] Rotavirus [79,80] Sin Nombre virus [81]
αVβ5	Kaposi’s sarcoma-associated herpesvirus [82] Adenovirus [78] Epstein-Barr virus [83]
αVβ6	Coxsackievirus A9 [73] Foot-and-mouth disease virus [84,85] Epstein-Barr virus [83] Herpes simplex virus [86]
αVβ8	Epstein-Barr virus [83] Herpes simplex virus [86]
αXβ2	Rotavirus [60]
αIIbβ3	Sin Nombre virus [81]

**Table 3:** Integrin binding by small molecules, hormones, growth factors, and venoms.

Integrin	Non-ECM Ligand	Function [Key Refs]
α1β1	KTS/RTS disintegrins	Block cell adhesion [128,129]
α2β1	EMS16 CLP	Block adhesion to collagen [130,131]
α3β1	VEGF Disintegrin Lebein 1/2	Cell adhesion [132] Block cell adhesion [133]
α4β1	MLD disintegrins	Block cell adhesion [128]
α4β7	MLD disintegrins	Block cell adhesion [128]
α5β1	ANGPTL2	Cancer cell migration/proliferation [134] Macrophage pro-inflammatory response [135]
α6β1	Disintegrin Lebein 1/2	Block cell adhesion [133]
α7β1	Disintegrin Lebein 1/2	Block cell adhesion [133]
α9β1	VEGF-A, -C, -D MLD disintegrins	Endothelial adhesion & lymphangiogenesis [136] Block cell adhesion [128]
αVβ3	Resveratrol Thyroid hormones (T3/T4) DHT ANGPTL3 ANGPTL4 VEGF	Anti-angiogenesis [137,138,139] Cell proliferation/angiogenesis [140,141,142] Cancer cell proliferation [143,144] Podocyte motility [145] Enhanced endothelial junctions [146] Endothelial cell adhesion [132]
αVβ5	ANGPTL4	Reduce proteinuria [147]
αVβ6	Pro-TGFβ	TGFβ activation [148,149]

Abbreviations: lysine-threonine-serine (KTS), arginine-threonine-serine (RTS), C-type lectin-like protein (CLP), vascular endothelial growth factor (VEGF), methionine-leucine-aspartic acid (MLD), angiopoietin-like protein (ANGPTL), dihydrotestosterone (DHT), transforming growth factor β (TGFβ)

**Table 4:** Integrin binding by bacteria and parasitic organisms.

Integrin	Species	Binding Protein [Key Refs]
α2β1	*Ancylostoma caninum*	Hookworm platelet inhibitor (HPI) [193,194]
αIIbβ3	*Ancylostoma caninum* *Macrobdella decora* *Tabanus yao* *Ornithodoros moubata* *Ixodes pacificus* *Dermacentor variabilis*	Hookworm platelet inhibitor (HPI) [193,194] Decorsin [195] Vasotab TY [196] Tablysin-15 [197] Disagregin [198] YY-39 [199] Variabilin [200]
α3β1	*Borrelia burgdorfori* *Yersinia*	BBB07, BB0172 [170] Invasin [187,188]
α4β1	*Escherichia coli* *Yersinia*	Intimin [157] Invasin [187,188]
α5β1	*Helicobacter pylori* *Escherichia coli* *Shigella flexneri* *Entamoeba histolytica* *Yersinia*	CagL [177,179] Intimin [157] Ipa B, C, D [158] EhCP5 [201] Invasin [187]
α6β1	*Yersinia*	Invasin [187,188]
αMβ2	*Bordetella pertussis* *Ancylostoma caninum*	Filamentous hemagglutinin protein [159] Neutrophil inhibitor factor (NIF) [202]
αVβ1	*Yersinia*	Invasin [187]
αVβ3	*Borrelia burgdorfori* *Helicobacter pylori* *Entamoeba histolytica*	P66 [165] CagL [177] EhCP5 [203,204]
αVβ5	*Helicobacter pylori*	CagL [178,180]
αVβ6	*Helicobacter pylori*	CagL [181]
